# Longitudinal changes in resting state networks in early presymptomatic carriers of C9orf72 expansions

**DOI:** 10.1016/j.nicl.2020.102354

**Published:** 2020-07-20

**Authors:** Rachel Smallwood Shoukry, Rebecca Waugh, Dan Bartlett, Denitza Raitcheva, Mary Kay Floeter

**Affiliations:** a National Institute of Neurological Disorders and Stroke, National Institutes of Health, Bethesda, MD, 10 Center Drive, 20892-1140, USA; b Biogen, 225 Binney Street, Cambridge, MA 02142, USA

**Keywords:** Functional connectivity, C9orf72, Amyotrophic Lateral Sclerosis, Presymptomatic mutation carriers, Longitudinal

## Abstract

Previous cross-sectional imaging studies found differences in brain structure and in resting state networks between presymptomatic carriers of mutations in C9orf72 (C9+) and healthy controls. We carried out a prospective longitudinal study of clinical and resting state functional imaging in a cohort of 15 presymptomatic C9+ carriers to determine whether differences in resting state connectivity prior to developing symptoms reflect static developmental differences or ongoing low-grade degenerative changes. Presymptomatic C9+ carriers were scanned at baseline with follow-up scanning at 6- and 18-months and compared to a cohort of 14 healthy controls scanned longitudinally. Resting state networks associated with manifest disease were visualized by comparing 27 symptomatic C9+ carriers to 34 healthy controls. Motor, salience, thalamic, and speech production networks were visualized using a seed-based analysis. Neurofilament light chain was measured in serum obtained at the time of the scans. Neither clinical measures of motor, cognitive, and behavioral function nor neurofilament levels changed over follow-up in presymptomatic C9+ carriers. In thalamic networks, there was a reduction in connectivity in presymptomatic carriers at all timepoints with a constant difference compared to healthy controls. In contrast, precuneus/posterior cingulate regions exhibited declining functional connectivity compared to controls over the 18-month follow-up, particularly in motor networks. These were regions that also exhibited reduced functional connectivity in symptomatic C9+ carriers. Reduced connectivity over time also occurred in small regions of frontal and temporal cortex within salience and thalamic networks in presymptomatic C9+ carriers. A few areas of increased connectivity occurred, including cortex near the motor seed and within the speech production network. Overall, changes in functional connectivity over time favor the explanation of ongoing low-grade alterations in presymptomatic C9+ carriers in most networks, with the exception of thalamic networks where functional connectivity reductions were stable over time. The loss of connectivity to parietal cortex regions in several different networks may be a distinct feature of C9orf72-related degeneration. Longitudinal studies of carriers who phenoconvert will be important to determine the prognostic significance of presymptomatic functional connectivity alterations.

## Highlights

• Functional connectivity changes over 18 months in presymptomatic *C9orf72* carriers.
• Thalamic networks have reduced functional connectivity, but are stable over time.
• Presymptomatic FC patterns trend toward those of symptomatic *C9orf72* carriers.
• Reduced connectivity of posterior parietal regions occurs in different networks.

## 1 Introduction

Carriers of mutations in genes that cause neurodegenerative disorders can be identified by genetic testing many decades before symptoms begin. A recent conceptual model proposed distinct stages within the presymptomatic phase of amyotrophic lateral sclerosis (ALS) (Benatar et al., 2019). These include a pre-manifest stage in which biomarkers of the disease process can be detected without clinical symptoms. In familial ALS, neurofilament proteins from degenerating axons can be detected in the spinal fluid or blood of presymptomatic carriers up to a year before clinical symptoms (Benatar et al., 2018, van der Ende et al., 2019), thus marking the onset of the pre-manifest stage. Theoretically, there could be novel biomarkers that detect changes that occur even prior to axonal breakdown, such as synaptic loss or changes in functional circuits and networks.

Repeat expansion mutations in the gene *C9orf72* are the most common genetic cause of ALS and frontotemporal dementia (FTD) in the United States. Symptomatic carriers can have varied amounts of motor and cognitive symptoms. Imaging studies of symptomatic carriers of *C9orf72* expansion mutations (hereafter referred to as C9+ carriers) show progressive global atrophy, loss of white matter integrity in frontal brain regions and the corticospinal tract, and reduced functional connectivity (Bede et al., 2013, Lee et al., 2014, Mahoney et al., 2015, Floeter et al., 2016, Floeter et al., 2018). Subtle structural and functional imaging differences have been reported between young adult presymptomatic C9+ carriers and familial non-carriers of similar age in group comparisons (Walhout et al., 2015, Lee et al., 2017, Papma et al., 2017, Cash et al., 2018, Wen et al., 2019). Presymptomatic C9+ carriers also were found to have lower, although not abnormal, scores on cognitive testing than familial non-carriers (Rohrer et al., 2015, Papma et al., 2017). It has been debated whether such cross-sectional differences arise during development or result from a slowly progressive disease process (Walhout et al., 2015, Lee et al., 2017, Caverzasi et al., 2019). One 2-year longitudinal imaging study did not detect progression of structural differences in presymptomatic C9+ carriers (Panman et al., 2019).

To explore how functional networks change during the presymptomatic phase of disease, we carried out a longitudinal clinical and imaging study of presymptomatic C9+ carriers. The networks examined – motor, salience, thalamic, and speech production – while not intended to represent an exhaustive list of affected networks or regions – were selected *a priori* based on the known clinical features of C9+ ALS and FTD patients and previous literature. Our working hypothesis was that presymptomatic carriers would exhibit changes in those networks known to be affected in symptomatic carriers. The motor network was selected because ALS affects the motor system and motor function. The salience network was selected due to literature findings of atrophy and altered functional connectivity in bvFTD (Filippi et al., 2013, Lee et al., 2014). We chose the thalamus based on previous literature that found structural and functional differences in C9+ carriers (Lee et al., 2014, Lee et al., 2017). The speech production network is known to exhibit alterations in ALS patients (Abrahams et al., 2004) and was selected because bulbar dysfunction is common in ALS. Goals of the study were 1) to determine whether functional connectivity differs between presymptomatic C9+ carriers and healthy controls in motor and non-motor networks, 2) to characterize the trajectory of functional connectivity differences over time in presymptomatic C9+ carriers, 3) to explore similarities of functional connectivity patterns between symptomatic and pre-symptomatic C9+ carriers, and 4) to correlate functional connectivity differences with clinical measures of motor function and cognitive-behavioral function.

## 2 Methods

### 2.1 Participants

MRI scanning was carried out on forty-two carriers of *C9orf72* expansion mutations (C9+ carriers) and thirty-four healthy subjects who gave written informed consent for protocols approved by the NIH CNS Institutional Review Board (NCT01925196 and NCT01517087). All C9+ carriers had >30 repeats by testing in CLIA-certified laboratories. The number of repeats was not further quantified. All but two C9+ carriers were unrelated (of whom one was presymptomatic). The C9+ carriers were enrolled in a prospective natural history study with longitudinal clinical and imaging measures at baseline, 6-months, and 18-months. In addition to a baseline scan, longitudinal MRI scans were obtained on thirty-two of the unrelated C9+ carriers, including 15 presymptomatic C9+ carriers, and twenty-two healthy subjects.

### 2.2 Clinical evaluation

All participants were examined by an experienced neurologist and diagnostic tests were performed to determine diagnosis. C9+ carriers who met revised El Escorial criteria for possible, probable, or definite ALS (Brooks et al., 2000) were classified as ALS. Electromyography was carried out on all C9+ carriers at the initial visit and at subsequent visits if they not already met criteria for a diagnosis of ALS. C9+ carriers who met consensus criteria for behavioral variant FTD (Rascovsky et al., 2011) were classified as FTD. Motor and cognitive testing was carried out on all C9+ carriers, including presymptomatic carriers, at each visit. Tests included the MiniMental State Examination (Folstein et al., 1975) as a measure of global cognition, Letter Fluency (written or oral) and Trails subtests of the Delis-Kaplan Executive Function System as measures of executive function (Delis et al., 2004), and the Mattis Dementia Rating Scale (DRS-2), which includes a memory subscore (Jurica et al., 2001). The Frontal Behavioral Inventory-ALS version (FBI) (Murphy et al., 2015) was administered to caregivers to assess behavioral impairment. Motor function was quantified at each visit using the ALSFRS-R (Cedarbaum et al., 1999) and measures of motor performance, including finger and foot tapping, 9-hole peg test, 25-foot timed gait, and timed reading of a standard passage (“My Grandfather” (Van Riper 1963)).

Human serum samples were tested using the Quanterix Simoa NF-Light kit on the Quanterix HD-1 instrument according to the manufacturer’s fully automated two-step immunoassay protocol (www.quanterix.com). Calibrator, control, and serum samples were loaded into the HD-1 instrument, where samples are mixed with NfL antibody beads and biotinylated detector antibody. Complexed samples were transferred to the Simoa Disc for measurement of fluorescence. The fluorescent signal generated was proportional to the amount of target analyte present in the sample, and the concentrations in the unknown samples were interpolated from the calibration curve. All specimens were run in a single batch using the same kit lot reagents.

All healthy controls had a normal neurological exam and were screened for cognitive impairment with the Montreal Cognitive Assessment (www.mocatest.org) and/or MiniMental State Examination at the first visit.

### 2.3 Imaging acquisition

**Supplementary figure 1:**
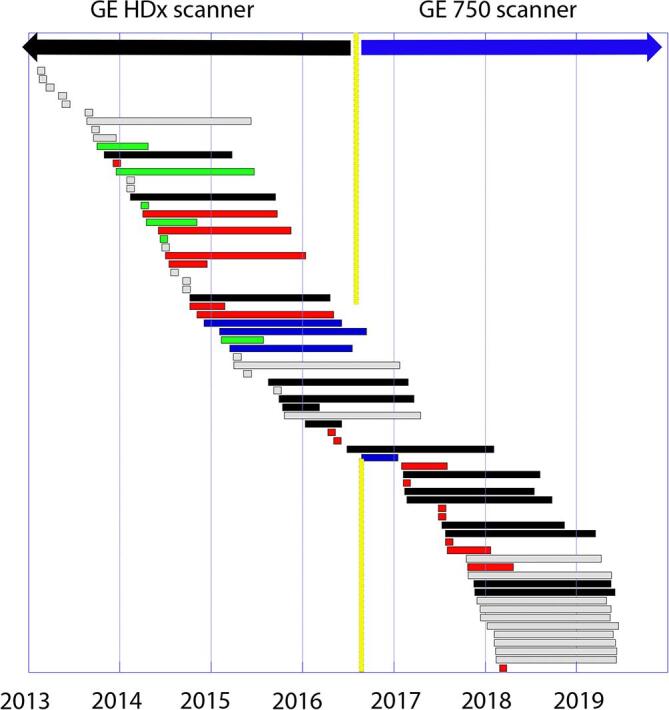
Timeline for acquisition of MRI scans for all subjects in this study. Grey=Healthy controls; Red = C9+ ALS; Green = C9+ ALS-FTD; Blue = Dementia; Black = C9+ presymptomatic carriers. The length of each bar represents the time from first to last scan acquired. Squares represent subjects with only a single scan. The yellow bar indicates the timing of the 3T scanner upgrade, from GE HDX to GE 750.

MRI scans were collected between 2012 and 2019 on 3T GE HDX and GE 750 scanners. Subjects from all three groups (symptomatic C9+ patients, presymptomatic C9+ carriers, and healthy controls) were enrolled throughout this period for the baseline scan, with follow-up scans scheduled for 6- and 18-months later. The timeline of scanning and distribution of subjects between scanners is shown in Supplementary Fig. 1. Scans included a high-resolution T1 scan for anatomical registration (Resolution 256 × 256, voxel size 1 × 0.938 × 0.938 mm, 176 slices) and a resting state functional MRI (TE/TR 30 ms/2s, FOV 240 × 240, Resolution 64 × 64, voxel size 3.75 × 3.75 × 3.8 mm, 40 slices). The rs-fMRI scan lasted approximately eight minutes with eyes open and fixation on a centered onscreen cross.

### 2.4 Image processing

Pre-processing steps were conducted with AFNI software (Cox, 1996) and included clipping the first four volumes, despiking, motion correction, spatial smoothing (FWHM kernel size = 5 mm) and intensity normalization.

White matter (WM), grey matter (GM) and cerebrospinal fluid (CSF) masks were derived by skull stripping and segmenting the anatomical T1 image. With these masks, mean functional activity time courses for each region in the resting state scan were generated. WM and CSF time courses, six motion parameters and their derivatives, and a high-pass temporal filter comprised the nuisance covariates in a regression model of the data, whose function was to estimate the normalized functional data without these sources of physiological noise (Birn, 2012). Each subject’s T1 image was registered to the MNI152 2 mm standard brain using a two-step process of affine alignment followed by non-linear warping. Inclusion of the affine alignment was particularly effective for symptomatic subjects with substantial atrophy.

Seeds were chosen to generate motor and thalamic networks bilaterally, the salience network, and a speech production network. Seed locations were positioned based on MNI coordinates as spheres with 5 mm radii, then converted to each subject’s native space with inverse transformation maps derived from warping the anatomical image to standard space. The MNI coordinates of the six seed used to generate intracortical resting state networks are shown in Table 1.

**Table 1:** Location of seeds used for generating resting state networks.

	MNI Coordinates	Network Seed Abbreviation
Right Motor Cortex	[42–16 52]	R_Motor
Left Motor Cortex	[−42 20 52]	L_Motor
Right Anterior Insula	[34 18 6]	Salience
Left Pars Opercularis	[−44 8 24]	Pars Op
Right Thalamus	[14 −29 2]	R_Thal
Left Thalamus	[−11 −28 4]	L_Thal

The voxelwise correlation (Pearson’s R) between the fully pre-processed functional data and each of the six seed regions generated a connectivity map in native space that was subsequently converted to standard space. To account for inter-scanner variance, the ComBat protocol was employed with subject group (C9+ symptomatic, C9+ presymptomatic, or healthy control), age, interscan interval, gender, and a subject motion parameter as covariates (https://github.com/Jfortin1/ComBatHarmonization). The ComBat protocol has been shown to reduce variance associated with scanner batch effects (Fortin et al., 2017, Fortin et al., 2018, Yu et al., 2018).

### 2.5 Image analysis

2.5.1 To visualize network changes associated with advanced disease, a cross-sectional comparison of the resting state networks derived from the six seeds of symptomatic C9+ carriers versus healthy controls was carried out, with age as a covariate. For this analysis the last scan obtained from each symptomatic carrier was used, when disease was most advanced. General linear tests derived from a group ANOVA model in AFNI (3dMVM, (Chen et al., 2014)) assessed both possible contrast directions between the groups. To determine any significant clusters across the GM-masked whole brain region, we applied a whole-brain voxel-wise threshold of p < 0.001 uncorrected to these statistical maps. AFNI’s 3dClustSim estimated the required size (k voxels, NN = 1) of clusters to achieve FWE-corrected significance thresholds of p < 0.05 and p < 0.10 for multiple comparisons. Cluster size is inversely related to the significance threshold, with larger clusters required for p < 0.05 than p < 0.10; thus clusters with a corrected p < 0.05 are referred to as “large clusters”, whereas those at p < 0.1 are referred to as “small clusters”. The voxel sizes for all clusters are provided in Table 3.

2.5.2 Longitudinal comparisons of resting state networks were carried out between presymptomatic C9+ carriers and a subset of the healthy controls more closely matched for age (N = 14, mean age at baseline = 47.7, 9 females). A linear mixed effects model (Chen et al., 2013) accounted for the longitudinal aspect of the model and allowed for missing data from three presymptomatic C9+ carriers who did not complete every scan. Age and interscan interval were included as covariates. Clusters of interest were defined using the same method as the cross-sectional model.

### 2.6 Statistics

Demographic and clinical data are shown in tables as mean ± standard deviation. Statistics were computed using GraphPad Prism (v. 8.3.1) and IBM SPSS (subscription Build 1.0.0.1347). Differences in clinical measures between the three groups were tested by ANOVA with post-hoc Tukey’s test to correct for multiple comparisons. Measures were tested for normality and an unpaired *t*-test or Mann-Whitney test was used to compare cognitive measures between symptomatic and presymptomatic C9+ carriers. Correlations between clinical measures and connectivity measures from clusters of interest were assessed with Pearson’s R using p < 0.05 as the threshold for significance.

## 3 Results

### 3.1 Demographics and clinical testing

Fifteen of the C9+ carriers were presymptomatic at their first visit, with normal neurological exams, EMGs, and cognitive testing. None of the presymptomatic C9+ carriers developed ALS or dementia during follow-up scanning. Phone follow-up continued for a year afterward, and no phenoconversions had occurred. Of the 27 symptomatic C9+ carriers, 17 had ALS, 6 had ALS-FTD, and 4 had dementia. Presymptomatic C9+ carriers were younger on average than symptomatic C9+ carriers and a greater proportion were women (Table 2). The age of the healthy controls was intermediate between the symptomatic and presymptomatic C9+ carriers. Tests of cognitive and motor function showed no differences between presymptomatic C9+ carriers and healthy controls. Symptomatic C9+ patients had impaired cognitive and motor testing compared to controls and presymptomatic C9+ carriers (Table 2). Among the symptomatic C9+ patients, ALSFRS-R, Trails, Fluency, Memory, and FBI scores differed among the three clinical diagnostic groups (ANOVA, p < 0.05). C9+ ALS patients had greater abnormalities on motor measures, whereas cognitive measures were more impaired in C9+ patients with FTD/Dementia (Supplemental Table 1).

**Table 2:** Demographic features and clinical measures of carriers of C9orf72 mutations and healthy controls.

	Healthy Controls	C9+ Symptomatic	C9+ Presymptomatic		
	(n = 34)	(n = 27)	Baseline (n = 15)	6-month follow-up (n = 15)	18-month follow-up (n = 12)
Age	51.4 ± 9.3	57.1 ± 9.8	43.4 ± 9.7^a^	43.9 ± 9.64	46.6 ± 9.89
% Male	53%	63%	20%	20%	25%
Symptom Duration (mos.)		36.6 ± 25.1			
Cognitive-Behavioral Domains					
MMSE	29.0 ± 1.0	26.9 ± 4.7^b^	29.1 ± 0.9	29.3 ± 0.9	29 ± 1.95
Trails B-A*(s)		111.1 ± 136.9^e^	35.9 ± 21	45.9 ± 29.9	30.2 ± 18.9
Letter Fluency (words/letter)	14.3 ± 3.6 (n = 22)	7.4 ± 4.1^b^ (n = 26)	12.7 ± 4.3	14.6 ± 4.3	15.1 ± 3.4
Memory (DRS raw score)		21.7 ± 4.9^f^	24.3 ± 1.0	24.2 ± 0.9	24.1 ± 1.0
Frontal Behavioral Inventory (% possible)		21.8 ± 23.7^e^	3.2 ± 4.6	2.2 ± 3.6	4.4 ± 12.0
Motor Domain					
ALSFRS-R	48	35.9 ± 7.3	47.9 ± 0.4	47.7 ± 0.6	47.3 ± 1.6
R Finger taps/10 s	62.6 ± 7.1	46.3 ± 13.4^b^ (n = 26)	58.2 ± 7.3	56.5 ± 7.8	56.7 ± 10.5
L Finger taps/10 s	58.2 ± 8.8	38.8 ± 15.5^b^ (n = 26)	56.2 ± 7.4	55.8 ± 9.7	54.4 ± 9.1
R foot taps/10 s	45.0 ± 6.4	29.0 ± 13.7^b^ (n = 24)	44.2 ± 6.1	44.1 ± 4.5	43.2 ± 4.4
L foot taps/10 s	41.9 ± 8.0	29.0 ± 13.9^b^ (n = 24)	42.4 ± 5.6	42 ± 4.8	40.6 ± 4.3
R 9-hole peg (s)	18.6 ± 2.9	40.5 ± 30.9^c^ (n = 26)	19.8 ± 2.6	19.8 ± 3.1	20.6 ± 3.0
L 9-hole peg (s)	19.4 ± 2.2	71.5 ± 120.7^c^ (n = 25)	20.3 ± 3.2	20.5 ± 3	19.6 ± 6.8
Gait 25ft (s)	4.2 ± 0.9	7.5 ± 2.5^d^ (n = 24)	6.0 ± 0.9	6.2 ± 1.1	6.3 ± 0.8
Reading Passage (s)	55.2 ± 13.8	82.9 ± 45.3^c^ (n = 20)	49.1 ± 11.5	48.2 ± 10.2	49.4 ± 6.9

a C9+ presymptomatic < HC and C9+ symptomatic, ANOVA p < 0.05 corrected.

b C9+ symptomatic < HC and C9+ presymptomatic, ANOVA p < 0.05 corrected.

c C9+ symptomatic > HC and C9+ presymptomatic, ANOVA p < 0.05 corrected.

d HC< C9+ presymptomatic > C9+ symptomatic, ANOVA p < 0.05 corrected.

e C9+ presymptomatic < C9+ symptomatic, *t*-test p < 0.05.

f not significant, Mann-Whitney test.

**Fig. 1:**
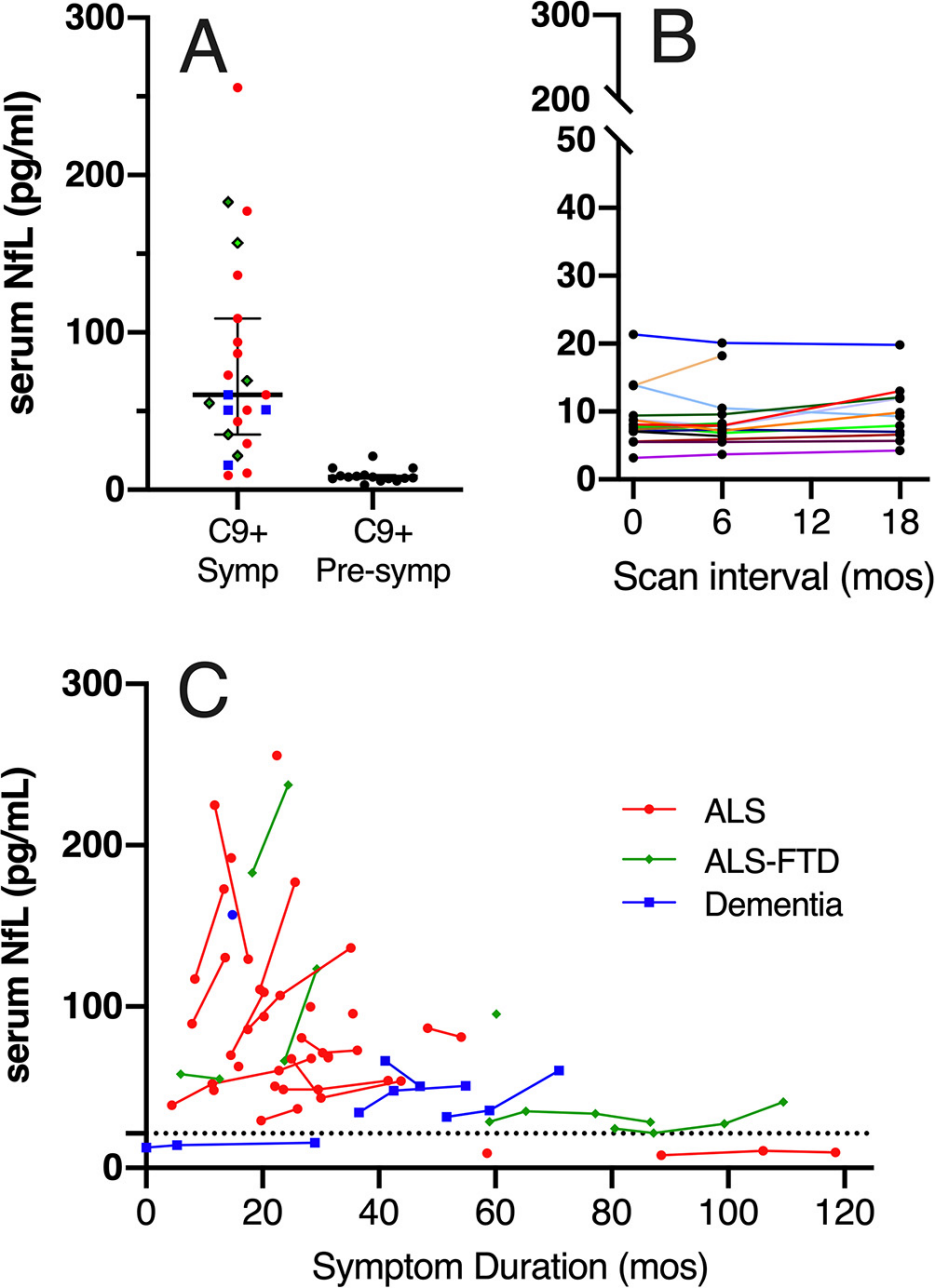
Levels of neurofilament light chain (NfL) in serum in A) Symptomatic *C9orf72* carriers (C9+ Symp) and presymptomatic *C9orf72* mutation carriers (C9+ Pre-symp) carriers at the time of scans. Symbols indicate clinical diagnoses red circles = ALS, green diamonds = ALS-FTD, blue squares = dementia. Median and interquartile range are shown. NfL levels shown for presymptomatic carriers in panel A were drawn at the time of the first scan. B) Longitudinal NfL levels in presymptomatic carriers. Each colored line represents a different presymptomatic carrier. C) Levels of NfL in symptomatic C9+ carriers in relation to symptom duration, showing high levels early in symptom onset. Each line indicates an individual C9+ patient (red circles = ALS, green diamonds = ALS-FTD, blue squares = FTD). The dotted line represents the highest serum NfL measure obtained in the presymptomatic C9+ carriers. (For interpretation of the references to colour in this figure legend, the reader is referred to the web version of this article.)

Asymptomatic C9+ carriers had lower levels of serum NfL compared to symptomatic C9+ carriers (Mann Whitney test p < 0.0001; Fig. 1A). Serum NfL levels remained low in presymptomatic C9+ carriers throughout the 18-month follow-up (Fig. 1B), in contrast to symptomatic C9+ carriers in whom rising levels often occurred in the months after the onset of symptoms (Fig. 1C).

### 3.2 Connectivity differences between healthy controls and symptomatic C9+ carriers

**Fig. 2:**
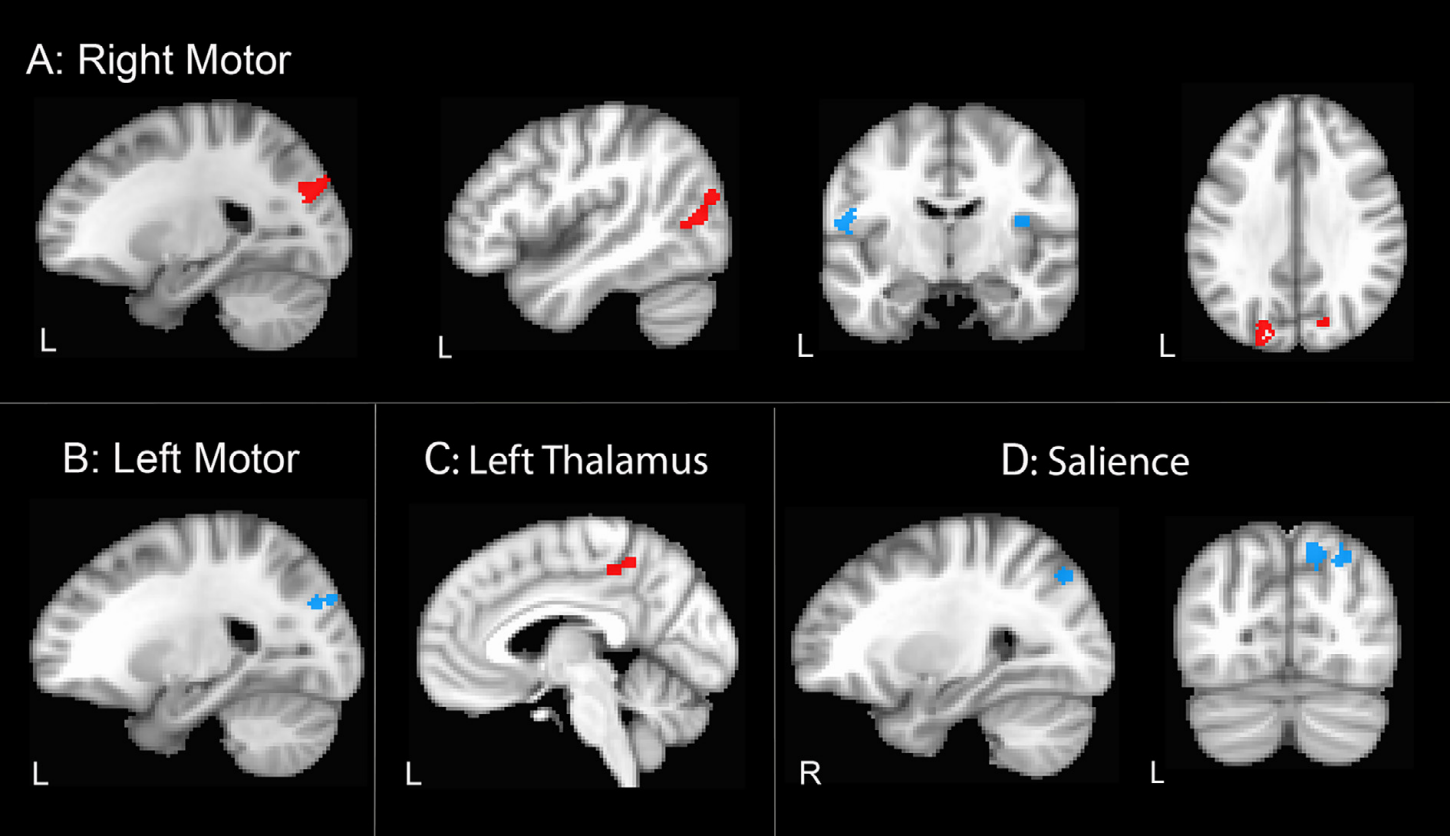
Regions with reduced functional connectivity in C9+ symptomatic C9+ carriers compared to healthy controls. A) Right motor network with five clusters (labeled CS2-6 in Table 3) B) Left motor network with 1 cluster (CS1), C. Left thalamus network with 1 cluster (CS9). D. Salience network with two clusters (CS7-8). Red indicates threshold p < 0.05 FWE-corrected; blue indicates threshold p < 0.1 FWE corrected. The hemisphere of sagittal slices is labelled L, left, or R, right. Note that the precuneus has reduced functional connectivity in both motor networks and the salience network. (For interpretation of the references to colour in this figure legend, the reader is referred to the web version of this article.)

Symptomatic C9+ carriers only had regions of decreased connectivity compared to healthy controls. There were no regions with increased connectivity. Clusters in five cortical regions had reduced functional connectivity with the right motor cortex seed, including three large clusters in the right and left precuneus and the middle temporal gyrus and two small clusters in the right insula and left precentral gyrus (Fig. 2A, Table 3A). The left motor seed also showed reduced connectivity to the left precuneus (Fig. 2B). The left precuneus also exhibited reduced functional connectivity with the left thalamus (Fig. 2C). Two small clusters in the right precuneus had reduced connectivity with the salience network seed (Fig. 2D).

**Table 3:** Clusters with differing functional connectivity in C9+ carriers compared to healthy controls.

Label	Seed	Contrast	Cluster Size (voxels)	Cluster focus MNI Coordinates	Cluster location	Cluster FWE-corrected p value
A: Cross sectional study of symptomatic C9orf72 carriers						
CS1	L Motor	HC > SxC9+	66	[−22,−80,32]	L Precuneus	p < 0.1
CS2	R Motor	HC > SxC9+	152	[−24,−80,32]	L Precuneus	p < 0.05
CS3	R Motor	HC > SxC9+	118	[45,−71,10]	R Middle Temporal	p < 0.05
CS4	R Motor	HC > SxC9+	115	[12,−87,42]	R Precuneus	p < 0.05
CS5	R Motor	HC > SxC9+	71	[42,−6,13]	R Ant Insula	p < 0.1
CS6	R Motor	HC > SxC9+	67	[−54,−8,16]	L Precentral, Postcentral	p < 0.1
CS7	Salience	HC > SxC9+	75	[14,−77,49]	R Precuneus	p < 0.1
CS8	Salience	HC > SxC9+	66	[27,−75,49]	R Precuneus, Sup parietal	p < 0.1
CS9	L Thal	HC > SxC9+	89	[−3,−43,53]	L Paracentral lobule, Precuneus	p < 0.05
B: Longitudinal analysis of presymptomatic C9orf72 carriers						
LG1	L Thal	HC > PreC9+	149	[4,−62,52]	R Precuneus	p < 0.05
LG2	R Thal	HC > PreC9+	532	[2,−54,33]	L & R Post Cingulate, L precuneus	p < 0.05
LG3	R Thal	HC > PreC9+	117	[−11,−28,35]	L Post Cingulate	p < 0.05
LG4	R Thal	HC > PreC9+	72	[−15,−48,44]	L Post Cingulate, Precuneus	p < 0.1
LG5	L Motor	HC > PreC9+ × time	341	[42,−62,32]	R Supramarginal, Angular, Inf Parietal, Superior Temporal	p < 0.05
LG6	L Motor	HC > PreC9+ × time	253	[6,−54,38]	R Precuneus, Cingulate	p < 0.05
LG7	Salience	HC > PreC9+ x time	65	[67,−34,−14]	R Middle Temporal	p < 0.1
LG8	L Thal	HC > PreC9+ × time	58	[7,25,34]	R cingulate, Medial Frontal	p < 0.1
LG9	L Motor	PreC9+ > HC	86	[−26,−97,1]	L Mid Occipital, Cuneus	p < 0.05
LG10	R Motor	PreC9+ > HC × time	82	[25,−23,58]	R Precentral	p < 0.05
LG11	Pars Op	PreC9+ > HC × time	221	[−38,−70,50]	L Inf parietal, Superior Parietal	p < 0.05

#### 3.2.1 Clinical correlates of functional connectivity changes in C9+ symptomatic carriers

In symptomatic C9+ carriers, the DRS-2 Memory score was modestly correlated with the connectivity between the right precuneus (cluster CS7) and the salience network seed (R = 0.408, p = 0.035). There were no significant correlations between the connectivity of any clusters and performance on executive function measures of Fluency and Trails, or MMSE, or FBI. Scores on several left-hand dexterity tasks exhibited correlations with clusters that had reduced connectivity in the right motor and left thalamus networks. Although the ALSFRS-R score was correlated with measures of motor function, it was not correlated with connectivity in any of the clusters (Supplemental Table 2).

### 3.3 Longitudinal differences between presymptomatic C9+ carriers and healthy controls

Although most regions had reduced or declining functional connectivity over time compared to controls, presymptomatic C9+ carriers exhibited a few regions with increased functional connectivity, in contrast to symptomatic C9+ carriers (Table 3B).

#### 3.3.1 Regions with decreased connectivity compared to controls

**Fig. 3:**
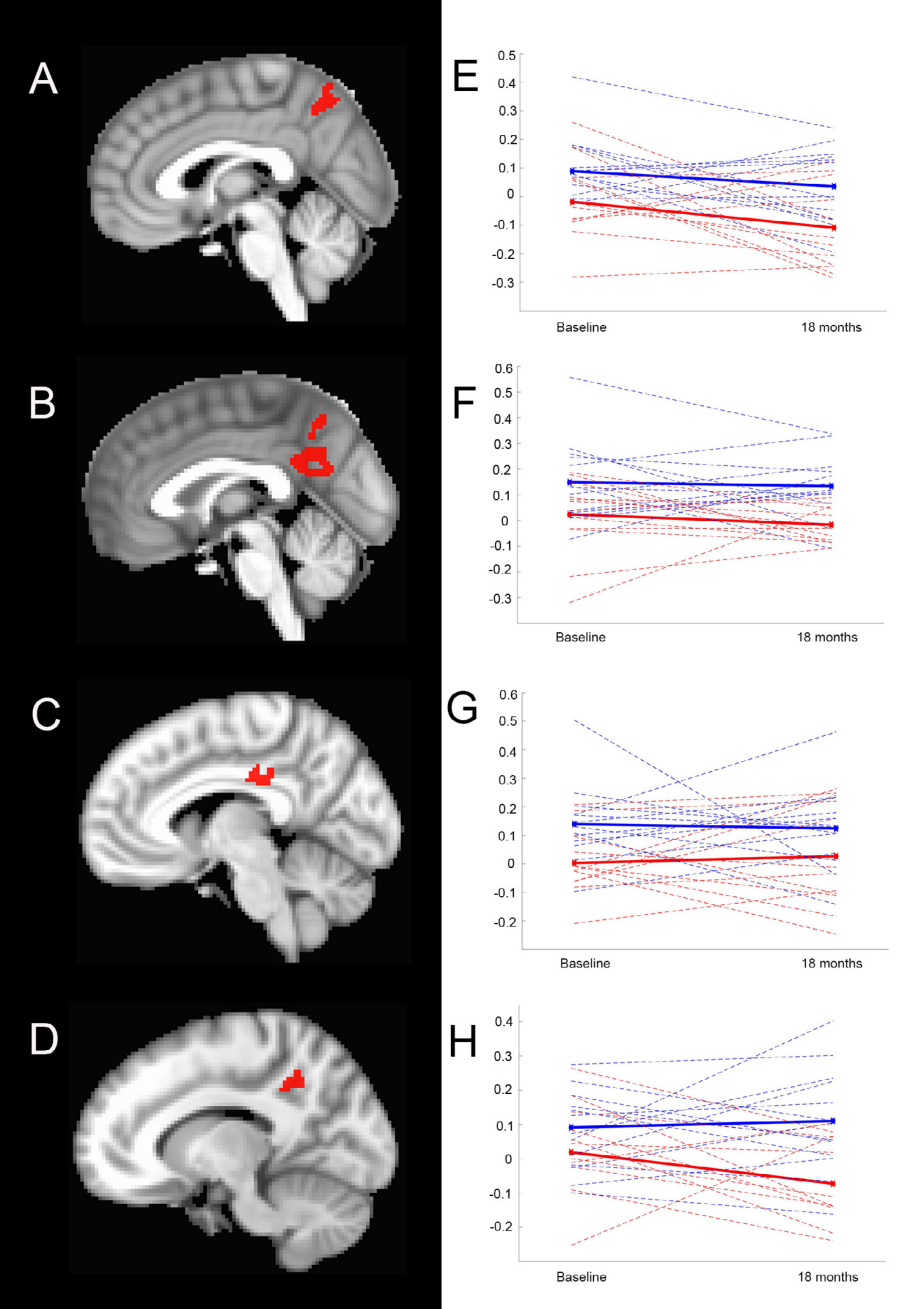
Regions with reduced functional connectivity in presymptomatic C9+ carriers with the left (A) and right (B-D) thalamus seeds that showed no difference longitudinally in slope of change compared to healthy controls. (E-H) Plots of mean functional connectivity (solid lines) for healthy controls (blue) and presymptomatic C9+ carriers (red) at baseline and 18 months for region in adjacent panel. Dotted lines show connectivity for individual participants. (For interpretation of the references to colour in this figure legend, the reader is referred to the web version of this article.)

Three nearly contiguous large clusters extending from the anterior precuneus to the posterior cingulate bilaterally had reduced connectivity in the thalamic networks (Fig. 3 A-D; Table 3B clusters LG1-4). However, the difference in thalamic connectivity remained constant over time from healthy controls (Fig. 3 E-H).

#### 3.3.2 Regions where connectivity decreased over time compared to controls

**Fig. 4:**
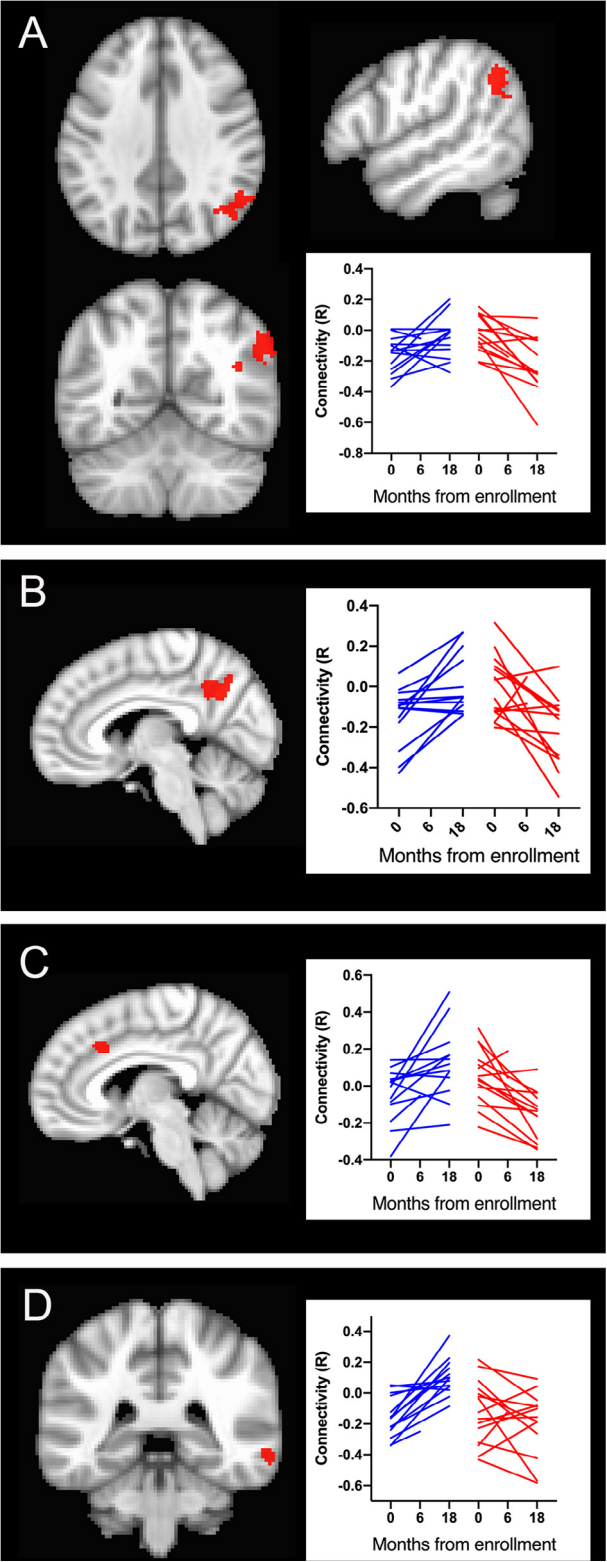
Regions in which functional connectivity declined over time in presymptomatic C9+ carriers compared to healthy controls. Plots within each panel show connectivity at baseline and 18 months for individual healthy controls in blue (left side) and presymptomatic C9+ carriers in red (right side) for regions illustrated in the panel. A) Axial, sagittal, and coronal views of a large region of parietal cortex with reduced functional connectivity to the left motor cortex seed (Cluster LG5, Table3B). B) Sagittal view of large region in precuneus with reduced functional connectivity to the left motor cortex seed (Cluster LG6, Table 3B). C) Small cluster in medial frontal cortex with reduced connectivity to left thalamus seed (Cluster LG8, Table 3B). D) Small cluster in mid-temporal cortex with reduced connectivity in the salience network (Cluster LG7, Table 3B). (For interpretation of the references to colour in this figure legend, the reader is referred to the web version of this article.)

Four clusters exhibited a pattern of decreasing functional connectivity over time which differed from the connectivity changes in healthy controls. The left motor network had two clusters with declining connectivity over time. One cluster consisted of a large region of the right posterior parietal cortex, encompassing the angular gyrus, supramarginal gyrus, and precuneus, and extending to the posterior cingulate (Fig. 4A, Table 3B cluster LG5). The other cluster was centrally located in the left precuneus (Fig. 4B, Table 3B cluster LG6). The remaining two clusters in which connectivity declined over time were small. A small cluster in the right medial frontal region (LG7) exhibited declining connectivity with the left thalamus seed (Fig. 4C). A cluster in in the right middle temporal cortex (LG8) exhibited declining connectivity with the salience network seed (Fig. 4D).

#### 3.3.3 Regions with increased and increasing connectivity

A large cluster in the left inferior parietal lobe had increased functional connectivity in the speech production network over time (Table 3B, cluster LG11). The other regions were more moderate in size, including a small cluster in the precentral gyrus with increased functional connectivity in the motor network (Table 3B, cluster LG10) and a cluster in the left occipital cortex with stably increased connectivity at to the motor network (Table 3B, cluster LG9).

#### 3.3.4 Clinical correlates of functional connectivity in presymptomatic C9+ carriers

The presymptomatic C9+ carriers had no significant decline in cognitive or motor clinical measures between their first and last scan. Because there was so little variability in clinical measures across sessions, there were few significant correlations between changes in scores and changes in connectivity. For a single timepoint, the last session, a lower letter fluency score was correlated with stronger connectivity of the cluster (LG11) in the left parietal cortex in the speech production network (r = -0.790, p < 0.001).

## 4 Discussion

We found that changes occurred in functional connectivity in carriers of *C9orf72* expansion mutations in the early presymptomatic period, without changes in biomarkers of axonal degeneration. The pattern of regions with reduced functional connectivity trended over time toward a similar pattern of reduced functional connectivity found in a cohort of symptomatic *C9orf72* mutation carriers, most of whom had ALS. Strikingly, several of the same parietal regions had reduced functional connectivity in different resting state networks. The precuneus and posterior cingulate had reduced functional connectivity in both motor and salience networks in symptomatic C9+ carriers compared to controls. These regions, as well as additional superior parietal regions, demonstrated reductions in thalamic and motor resting state networks in presymptomatic C9+ carriers. However, reduced connectivity emerged at different times in different networks of presymptomatic C9+ carriers. In thalamic networks, functional connectivity of the precuneus was lower than for healthy controls at the time of their first scan and remained stably lower over the 18 months of follow-up. In contrast, in the motor network, functional connectivity of precuneus/parietal cortex declined over follow-up. The early presymptomatic involvement of the thalamus in C9+ carriers is congruent with previous structural imaging studies (Walhout et al., 2015, Lee et al., 2017, Papma et al., 2017, Cash et al., 2018, Wen et al., 2019) and functional imaging in presymptomatic C9+ carriers (Lee et al., 2017). Our longitudinal findings would be consistent with the interpretation of a difference arising during development. Atrophy of the thalamus and posterior parietal cortex has been identified as part of the anatomical signature of both C9+ ALS (Bede et al., 2013, Agosta et al., 2017) and C9+ FTD (Lee et al., 2014). The pattern of connectivity changes in C9+ carriers differs from the pattern of sporadic ALS (Agosta et al., 2013, Heimrath et al., 2014, Schulthess et al., 2016), presymptomatic SOD1+ carriers (Menke et al., 2016), and the pattern in C9-negative FTD (Lee et al., 2014). It is not strictly a mixture of the connectivity changes of ALS and FTD. In C9+ carriers, the involvement of posterior parietal structures is a distinct feature.

In the parietal lobe, the precuneus has distinct subregions involved in sensorimotor integration, episodic memory, and visuospatial processing (Cavanna and Trimble, 2006). The anterior portion is involved in imagery of the self and, with the posterior cingulate, is considered to be a core region of the default mode network (DMN) (Cavanna and Trimble, 2006, Cunningham et al., 2017). The posterior portion of the precuneus is activated during episodic memory retrieval in the fronto-parietal network (Cavanna and Trimble, 2006) and during motor imagery (Hanakawa et al., 2003). Functional MRI studies support the proposition that the posterior precuneus is involved in a memory network distinct from the DMN (Deng et al., 2019). Differential involvement of the more posterior portion of the precuneus in C9+ carriers may resolve the apparent contradiction within literature reports that reduced salience network connectivity and increased DMN connectivity characterize FTD, whereas reduced connectivity in the DMN characterizes Alzheimer disease (Zhou et al., 2010). Of note, however, one study found that whereas increased DMN connectivity was found in FTD patients without *C9orf72* mutations, C9+ FTD had no change in DMN connectivity (Lee et al., 2014). We did not specifically assess the DMN or precuneus network in this study, but the overlap with regions showing decreased connectivity in C9+ carriers warrants further investigation. Although clinical findings of parietal dysfunction have been noted in symptomatic C9+ carriers (Majounie et al., 2012), a limitation of our study is that the clinical test battery focused on motor and executive function, rather than parietal function. We did find a correlation between memory scores and functional connectivity of precuneus in the salience network in symptomatic C9+ carriers.

Whereas symptomatic C9+ carriers only had regions of decreased connectivity in the resting state networks studied, presymptomatic C9+ carriers had a few regions with increased functional connectivity. The most notable was the increase in connectivity between the speech production network, from the seed in Broca’s area, and the inferior parietal cortex. Paradoxically, stronger connectivity in this network correlated with lower letter fluency scores. The interpretation of hyperconnectivity and why it would be associated with worse performance is uncertain. Increased functional connectivity could be produced by dysfunction of cortical inhibitory interneurons, leading to reduced selectivity of cortical activation as has been proposed in motor systems (Beck and Hallett, 2011, Mohammadi et al., 2011). Less selective activation in speech production networks could reduce efficient word retrieval. The lack of other correlations between connectivity strength and clinical measures in the presymptomatic C9+ carriers was not surprising as they showed no decline in cognitive or motor test measures over the observation period to suggest early phenoconversion.

Although many of our findings are consistent with previous cross-sectional resting state studies of C9+ presymptomatic carriers (Lee et al., 2017, Premi et al., 2019), we identified fewer and less extensive regions in which functional connectivity differed from healthy controls than other studies. In part, this is due to methodological differences, from different methods of acquisition (e.g. eyes open versus closed) and analysis (seed based versus independent components analysis). Given the relatively small sample size, we chose to use a seed-based approach to identify a few resting state networks, selecting seed regions based on prior literature and networks likely to be responsible for the most prominent clinical features in symptomatic C9+ carriers. If larger cohorts of presymptomatic C9+ carriers are imaged longitudinally in the future, methods for assessing whole brain functional connectivity, such as independent components analysis, may reveal other network alterations that our methods would not detect. Our analysis, applying a voxelwise statistical threshold followed by the determination of statistically significant cluster sizes, highlights statistical differences with fairly high stringency. As in other studies, we also saw considerable variability between individual carriers, as seen in our figures showing longitudinal plots of individuals. Additionally, the healthy controls in this study were not related to the C9+ carriers, and it is possible that using familial non-carrier controls would reduce variability sufficiently to allow additional differences to emerge in C9+ carriers. This study was limited to assessing functional connectivity, which has limitations as a single imaging modality for detecting individual differences. A multimodal approach to longitudinal studies will be needed to delineate the temporal relationship between functional connectivity and structural changes as well as fluid biomarkers. Although the longitudinal levels of serum NfL remained low in presymptomatic carriers, in contrast to symptomatic carriers, healthy control sera were not assayed in this batch, limiting conclusions regarding whether presymptomatic carriers sustain low-level axonal degeneration.

Whether familial neurodegenerative diseases begin slowly with a long period of low-grade, subclinical degeneration or more abruptly is a matter of debate. Under the recently proposed framework for presymptomatic ALS research (Benatar et al., 2019), the presymptomatic C9+ carriers in this study appeared to be in a very early stage, and levels of biomarkers of axonal degeneration remained low. Changes in functional connectivity progressed even during this early stage, favoring the idea that low- grade, subclinical changes occur over a substantial period. The mechanisms that could give rise to changes in circuit function, such as changes in synaptic strength or plasticity (Benussi et al., 2016), may be more amenable to intervention than later stages after neuronal loss begins. Finding that C9+ carriers remain asymptomatic despite reduced connectivity is consistent with a graph theory analysis that reported that the efficiency of intracortical networks remains resilient to declining functional connectivity and focal atrophy until just prior to the onset of clinically manifest disease in carriers of FTD genes (Rittman et al., 2019). Because the average age of our presymptomatic cohort was a decade younger than that of the symptomatic cohort, longer follow-up will be needed to determine whether the functional connectivity changes observed here are predictive of disease onset or allow identification of C9+ carriers at an earlier stage of disease.

## 5 Conclusions

The motor, salience, and thalamic resting state networks of *C9orf72* carriers differ from those of healthy controls prior to development of clinical symptoms and when levels of a serum marker of axonal degeneration remain low. Changes in networks of presymptomatic C9+ carriers occurred over 18 months of follow-up, with the predominant change being a decline in functional connectivity, particularly in motor networks. The precuneus region of the posterior parietal cortex emerged as a region with reduced connectivity in several networks. In presymptomatic C9+ carriers, the pattern that emerged over time began to resemble the pattern of reduced functional connectivity in symptomatic *C9orf 72* carriers.

## CRediT authorship contribution statement

**Rachel Smallwood Shoukry:** Methodology, Investigation, Writing - review & editing. **Rebecca Waugh:** Methodology, Software, Formal analysis, Writing - review & editing. **Dan Bartlett:** Investigation, Visualization, Writing - review & editing. **Denitza Raitcheva:** Resources, Writing - review & editing. **Mary Kay Floeter:** Conceptualization, Supervision, Resources, Writing - original draft.

## Declaration of Competing Interest

The authors declare that they have no known competing financial interests or personal relationships that could have appeared to influence the work reported in this paper.
