# Supplementary material for: Longitudinal changes in resting state networks in early presymptomatic carriers of C9orf72 expansions
Source: Neuroimage Clin. 2020 Jul 20;28:102354. doi: 10.1016/j.nicl.2020.102354 (PMC7406915; doi:10.1016/j.nicl.2020.102354)
Supplement: Supplementary data 1 [file mmc1.docx]

**Supplemental Table 1**. Clinical features of symptomatic C9+ patients by diagnostic group

|  |  | **C9+ symptomatic diagnostic groups** | | |
| --- | --- | --- | --- | --- |
|  | **Healthy Controls** | **C9+ ALS** | **C9+ ALS-FTD** | **C9+ Dementia** |
| N | 34 | 17 | 6 | 4 |
| Age | 51.4 ± 9.3 | 54.3 ± 9.4 | 59.9 ± 10.2 | 64.9 ± 7.0 |
| % Male | 53% | 47% | 100% | 75% |
| Motor symptom duration (mos) |  | 32.9 ± 23.0 | 16.2 ± 7.51 |  |
| Cognitive symptom duration (mos) |  |  | 40.0 ± 33.4 | 47.3 ± 23.1 |
| Cognitive-Behavioral Domains |  |  |  |  |
| MMSE | 29.0 ± 1.0 | 28.5 ± 1.6 | 24.5 ± 4.0 | 23.8 ± 10.6 |
| ***Trails B-A (s)** |  | 67.2 ± 45.5 | 141.5 ± 61.0 | 252 ± 325.4 |
| ***Letter Fluency (words/letter)** | 14.3 ± 3.6 (n=22) | 9.7 ± 3.0 | 3.3 ± 2.1 | 3.8 ± 2.6 |
| ***Memory (DRS raw score)** |  | 23.9 ± 1.5 | 20.0 ± 5.1 | 15.3 ± 8.0 |
| ***Frontal Behavioral Inventory (% possible)** |  | 8.8 ± 10.5 | 32.4 ± 11.5 | 61.3 ± 28.4 |
| Motor Domain |  |  |  |  |
| ***ALSFRS-R** | 48 | 33.4 ± 7.5 | 38.5 ± 3.9 | 42.5 ± 5.2 |
| R Finger taps/10s | 62.6 ± 7.1 | 43.4 ± 16.0  (n=16) | 50.2 ± 5.0 | 51.7 ± 6.7 |
| L Finger taps/10s | 58.2 ± 8.8 | 33.3 ± 16.8  (n=16) | 47.2 ± 8.6 | 48.4 ± 6.7 |
| R foot taps/10s | 45.0 ± 6.4 | 24.9 ± 11.5  (n=14) | 30.2 ± 17.0 | 41.3 ± 9.6 |
| L foot taps/10s | 41.9 ± 8.0 | 24.3 ± 12.3  (n=14) | 32.2 ± 15.5 | 40.6 ± 10.7 |
| R 9-hole peg (s) | 18.6 ± 2.9 | 48.4 ± 37.4  (n=16) | 28.4 ± 6.2 | 27.0 ± 7.2 |
| L 9-hole peg (s) | 19.4 ± 2.2 | 100.7 ± 150.5  (n=15) | 26.4 ± 7.2 | 29.7 ± 8.9 |
| Gait 25ft (s) | 4.2 ± 0.9 | 8.0 ± 2.6  (n=14) | 6.8 ± 2.0 | 6.9 ± 3.3 |
| Reading Passage (s) | 55.2 ± 13.8 | 86.7 ± 49.6  (n=11) | 85.5 ± 44.8  (n=5) | 69.4 ± 42.5 |

* significant difference between C9+ subtypes (p< 0.05, ANOVA, main effect of diagnosis group)
