# Supplementary material for: Longitudinal changes in resting state networks in early presymptomatic carriers of C9orf72 expansions
Source: Neuroimage Clin. 2020 Jul 20;28:102354. doi: 10.1016/j.nicl.2020.102354 (PMC7406915; doi:10.1016/j.nicl.2020.102354)
Supplement: Supplementary data 2 [file mmc2.docx]

Suppl Table 2. Correlations between clinical measures and functional connectivity of clusters exhibiting decreased connectivity in **symptomatic C9orf72 mutation carriers compared to healthy controls (Pearson’s R)**
